# From meal to malfunction: exploring molecular pathways, biomarkers and interventions in postprandial cardiometabolic health

**DOI:** 10.3389/fcvm.2025.1655889

**Published:** 2025-10-29

**Authors:** Claudia Reytor-González, Emilia Cevallos-Fernández, Belén Jácome, Daniel Simancas-Racines

**Affiliations:** ^1^ Universidad UTE, Facultad de Ciencias de la Salud Eugenio Espejo, Centro de Investigación en Salud Pública y Epidemiología Clínica (CISPEC), Quito, Ecuador; ^2^ Universidad UTE, Facultad de Ciencias de la Ingeniería e Industrias, Centro de Investigación de Alimentos (CIAL), Quito, Ecuador

**Keywords:** postprandial dysmetabolism, metaflammation, insulin resistance, cardiometabolic health, precision medicine, healthcare

## Abstract

Cardiometabolic diseases—including type 2 diabetes, cardiovascular disease, and metabolic dysfunction–associated steatotic liver disease—are increasingly driven by near-continuous after-meal exposure to glucose and lipid surges that traditional fasting tests often miss. This review prioritizes human studies from 2020 to 2025 and uses earlier work only as foundational anchors; non-English reports were excluded and preclinical findings are cited solely for mechanistic context. Evidence converges on six processes that amplify risk within hours after eating: impaired insulin signaling, delayed clearance of dietary lipids, mitochondrial and oxidative stress, loss of endothelial nitric oxide, inflammasome-mediated inflammation, and microbiome–hormone interactions. Dynamic, after-meal markers and simple composites such as the triglyceride–glucose index outperform fasting measures for identifying risk and guiding care. Practical strategies to shorten the “damage window” include Mediterranean-style meals with low glycemic index swaps and unsaturated fats, earlier distribution of daily energy and early time-restricted eating, a small pre-meal protein portion, and brief post-meal walking. Fast-acting medicines—glucagon-like peptide 1 and glucose-dependent insulinotropic polypeptide receptor agonists, rapid-acting insulin analogues, sodium–glucose cotransporter 2 inhibitors taken before meals, and proprotein convertase subtilisin/kexin type 9 inhibitors—further blunt peaks, while continuous glucose monitoring with algorithmic feedback enables timing-aware, person-specific adjustments. A tiered workflow—screen, stratify, and personalize—reframes prevention and treatment around after-meal physiology, with particular relevance to settings where resources are limited.

## 1 Introduction

Chronic noncommunicable diseases (CNCDs) now account for 41 million deaths each year, roughly 71 percent of all global mortality—and have overtaken infectious illnesses as the leading public-health threat (1). Within that broad category, cardiometabolic conditions—type 2 diabetes (T2D), cardiovascular disease (CVD) and metabolic-dysfunction-associated steatotic liver disease (MASLD, formerly NAFLD)—are rising fastest (2). T2D prevalence in sub-Saharan Africa has jumped from four million cases in 1980 to 23.6 million in 2021 and is projected to exceed 54 million by 2045 (3). MASLD affects roughly one adult in four worldwide (4, 5), while CVD alone claims 17.9 million lives annually, most of them in low- and middle-income regions (6).

Decades of epidemiology and mechanistic work converge on a common upstream driver: modern eating patterns characterized by frequent snacking on energy-dense, highly refined foods. This dietary behavior shortens fasting intervals and maintains most individuals in a near-continuous postprandial state—typically involving four to ten eating occasions per day with minimal overnight respite (7). These repeated surges of glucose and triglyceride-rich lipoproteins (TRLs) disrupt circadian clocks, overload mitochondrial redox systems, and activate innate-immune pathways, thereby accelerating atherogenesis, β-cell failure and hepatic steatosis (8, 9). Critically, this shift represents a departure from evolutionary eating patterns, where extended fasting periods allowed metabolic recovery and cellular repair processes that are now chronically interrupted.

The postprandial window now stretches well beyond half of every 24 h cycle; in many individuals it exceeds sixteen hours (10). Prolonged exposure to elevated glucose and lipid concentrations fuels low-grade systemic inflammation, a process termed “metaflammation”, which is central to the pathogenesis of T2D, CVD and MASLD (11, 12). The term postprandial dysmetabolism denotes the triad of hyperglycemia, hypertriglyceridemia, and hyperinsulinemia that follows each meal in susceptible individuals (13). When that triad is amplified by poor diet quality and increased meal frequency, oxidative stress, endothelial dysfunction and chronic inflammation ensue (14–16). Importantly, these metabolic perturbations can occur while fasting markers remain normal, highlighting a critical blind spot in current diagnostic approaches.

Prospective cohort studies demonstrate that the height and duration of post-meal glucose and triglyceride peaks predict carotid-intima thickening and future cardiovascular events even when fasting markers remain within normal ranges (17, 18). This finding challenges the traditional paradigm of metabolic assessment and underscores the clinical relevance of postprandial monitoring. Because the gut, liver, muscle, adipose tissue and pancreas coordinate postprandial homeostasis through complex inter-organ crosstalk, disturbances in any single organ rapidly propagate across the entire metabolic network (10, 17, 18). Despite this evidence, preventive care continues to rely predominantly on fasting glucose or low-density-lipoprotein cholesterol (LDL-C) measurements, leaving a substantial portion of cardiometabolic risk undetected and unaddressed—particularly in resource-limited settings where pharmacotherapy access is constrained and health-system capacity is limited (19–23).

This review applies a contemporary lens (2020–2025) reflecting methodological and clinical inflection points—widespread continuous glucose monitoring (CGM), standardized assays for TRL, multi-omics workflows, and the clinical introduction of glucagon-like peptide-1/glucose-dependent insulinotropic polypeptide (GLP-1/GIP) co-agonists—while selectively incorporating pre-2020 “foundational” contributions limited to seminal meta-analyses, consensus statements, pivotal randomized trials, or first-in-field mechanistic studies. Primary evidence prioritizes human adult studies indexed in Scopus (randomized controlled trials, controlled feeding/postprandial challenge studies over 0–6 h, and prospective cohorts), with inclusion contingent on clear test-meal composition, defined sampling windows, and assay standardization. Preclinical studies (animal or cell preparations) are cited only for mechanistic context, to probe causal links impractical or unethical to test in humans, and to nominate druggable targets relevant to the postprandial state [e.g., NADPH-oxidase (NOX)–endothelial nitric oxide synthase (eNOS) coupling, Yes-associated protein/TEA domain transcription factor (YAP/TEAD) signaling, calciprotein particle–driven pathways]. Such findings are not used to claim clinical efficacy, estimate effect sizes, or define clinical endpoints and are explicitly flagged in-text as “preclinical”, with model (mouse/rat) and exposure type (dietary, genetic, pharmacological) specified. We excluded non-English publications and did not treat narrative reviews as primary evidence; when cited, such reviews provided historical framing or methodological context only. Foundational citations are flagged in-text and collated in Supplementary Table S1 with rationale and study type (meta-analysis, pivotal RCT, first-in-field).

The aim of this narrative review is to move the spotlight from static fasting metrics to the dynamic metabolic stresses that arise after every meal, offering clinicians, researchers, and policymakers a practical roadmap for earlier detection, tailored intervention, and, ultimately, more effective prevention of CNCD-related morbidity and mortality.

This review synthesizes evidence on five inter-related domains of postprandial dysmetabolism: (i) the molecular and physiological pathways that precipitate metabolic dysfunction following nutrient intake; (ii) fasting-state surrogates and dynamic biomarkers that reveal these otherwise occult perturbations; (iii) dietary, behavioral, and pharmacological interventions that can mitigate postprandial stress; (iv) emerging technologies for real-time monitoring and personalized therapeutic targeting; and (v) implementation strategies for translating these advances into clinical practice, particularly in diverse populations and resource-variable settings.

## 2 Mechanistic drivers of postprandial dysmetabolism

### 2.1 Conceptual framework and temporal dynamics

Postprandial dysmetabolism is a time-dependent systems disturbance with min-to-hours fluctuations in glucose and lipids and hours-to-days adaptations in redox/circadian and gut–hormone axes. It reflects the convergence of nutrient overload, redox imbalance, and circadian misalignment across six interconnected nodes—from rapid glucose handling (min) to lipid clearance (peaks approximately 4–6 h) and microbiome–endocrine shifts (hours–days). Epidemiologic and clinical evidence links this state to endothelial injury and higher cardiovascular risk in people with and without T2D, supporting assay/intervention timing by temporal bands (operational definitions in Supplementary Table S1) (24, 25).

### 2.2 Substrate-specific metabolic overload (0–2 h post-meal)

Excess glucose engages canonical insulin signaling [insulin receptor substrate (IRS)—phosphoinositide 3-kinase (PI3K)—protein kinase B (Akt)] to drive glucose transporter type 4 (GLUT4) translocation in skeletal muscle and adipose tissue; impaired signaling delays vesicle delivery and prolongs hyperglycemia (26). Preclinical data indicate that SHIP2 (“SKIP” in rodents) limits phosphatidylinositol-3,4,5-trisphosphate (PIP3)/Akt signaling and that glucolipotoxic stress induces IRS-1 serine phosphorylation, dampening PI3K/Akt activity and GLUT4 trafficking (27–29). In parallel, intestinal chylomicron export can exceed lipoprotein lipase (LPL) capacity, leaving triglyceride-rich remnants that typically peak approximately 4–6 h (and may persist longer) after a mixed meal (30). The combined substrate surplus elevates mitochondrial reactive oxygen species (ROS) [reverse electron transport (RET) at Complex I; high potential at Complex III] within approximately 60–180 min, taxing antioxidant defenses (mechanistic/preclinical) (31, 32).

### 2.3 Vascular and inflammatory cascade (1–6 h post-meal)

The oxidative burst plus remnant lipoproteins activates the endothelium, lowers bioavailable nitric oxide (NO) (eNOS uncoupling; NOX/xanthine oxidase), and upregulates intercellular adhesion molecule 1/vascular cell adhesion molecule-1 (ICAM-1/VCAM-1). Innate sensors (Toll-like and NOD-like receptors) promote NOD-, LRR-, and pyrin-domain–containing protein 3 (NLRP3) inflammasome assembly, raising interleukin 1β (IL-1β) and interleukin 6 (IL-6); obesity amplifies these inputs via adipose-derived cytokines and lipotoxic mediators, reinforcing a feed-forward loop (33–35).

### 2.4 Microbiome-Endocrine integration (hours to days)

Dysbiosis reshapes the bile-acid pool via microbial bile-salt hydrolase (BSH) activity, modulating farnesoid X receptor/Takeda G-protein-coupled receptor 5 (FXR/TGR5) signaling (preclinical), and microbiota-derived bile acids/short-chain fatty acids (SCFAs) can influence L-cell GLP-1 secretion, helping explain variability in subsequent postprandial responses (foundational preclinical listed in Supplementary Table S1; contemporary human/preclinical syntheses) (36–38).

### 2.5 Clinical relevance and paradigm implications

**Figure 1:**
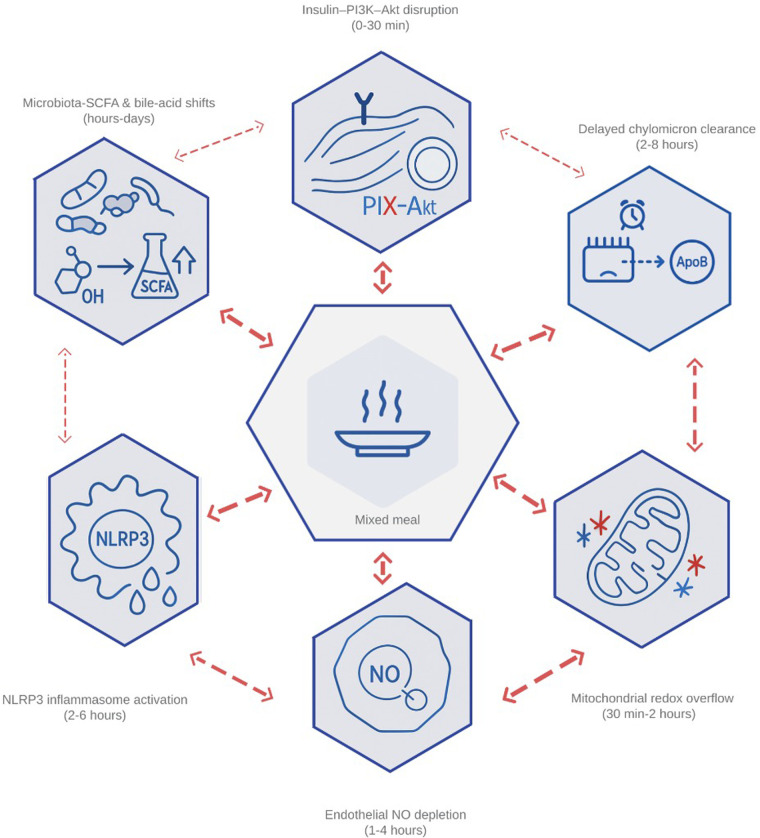
Integrated network of the six primary drivers of postprandial dysmetabolism. The diagram illustrates how (1) impaired insulin–PI3K–Akt signaling, (2) delayed clearance of chylomicron-derived remnants, (3) mitochondrial redox overflow, (4) endothelial nitric-oxide depletion, (5) inflammasome-driven cytokine release, and (6) microbiota-mediated shifts in bile-acid and short-chain-fatty-acid profiles interact within min after a mixed meal. Bidirectional arrows highlight feed-forward loops—ROS amplifying endothelial activation, remnant lipids fueling NLRP3 assembly, and butyrate modulating GLP-1—that transform transient surges into chronic cardiometabolic stress. PI3K, phosphoinositide-3-kinase; Akt, protein kinase B; ROS, reactive oxygen species; NLRP3, NOD-, LRR-, and pyrin-domain–containing protein 3 (inflammasome); GLP-1, glucagon-like peptide 1; SCFA, short-chain fatty acid; NO, nitric oxide.

Together, these nodes explain why the height and duration of post-meal peaks predict carotid-intima thickening and incident cardiovascular events independent of fasting markers (39, 40). With this theoretical framework, postprandial metabolism can be rapidly identified for targeted intervention; Figure 1 represents the interconnected network that links high nutrient intake with endothelial damage, insulin insensitivity, and hepatic lipid accumulation.

### 2.6 Glucose metabolism and insulin resistance

#### 2.6.1 Normal postprandial insulin signaling

In metabolically healthy adults, a meal elicits insulin secretion within 5–10 min. Circulating insulin binds to the insulin receptor (IR) in target tissues, resulting in autophosphorylation of the receptor as well as phosphorylation of IRS-1/2. IRS-1/2 recruits PI3K, generating PIP3 that recruits Akt to the membrane, where phosphoinositide-dependent kinase 1 and mammalian target of rapamycin Complex 2 (mTORC2) activate Akt (39, 40).

#### 2.6.2 Glucose uptake and metabolic integration

Akt phosphorylation of AS160 relieves Rab-GTPase restraint and drives GLUT4 vesicle fusion with the plasma membrane, enabling rapid glucose uptake. In parallel, Akt inhibits glycogen-synthase-kinase-3β to promote glycogen synthesis and—via mTORC1—supports protein synthesis and cell growth. Energy-sensing by adenosine monophosphate-activated protein kinase (AMPK) complements this program by enhancing GLUT4 trafficking and fatty-acid oxidation when the adenosine monophosphate/adenosine triphosphate (AMP/ATP) ratio rises, sustaining postprandial metabolic flexibility (41, 42).

#### 2.6.3 Temporal dynamics and individual variation

Nonetheless, postprandial glucose clearance varies substantially with age, fitness, genetics, and meal timing. In PREDICT 1, glycemic responses to identical meals showed large between-person differences with strong person-specific predictability (r = 0.77) (mixed-risk adults; *n* ≈ 1,100; standardized test meals approximately 500–900 kcal with varied macronutrient composition; capillary glucose/CGM sampling 0–4 h) (43). Variants at circadian loci (MTNR1B rs10830963, CRY2 rs12419690) relate to diurnal glycemic control (UK Biobank adults; *n* ≈ 420,000; random serum glucose linked to time-of-day; replication Estonian Biobank *n* approximately 100,000; 24 h cosinor modeling; not a meal test) (44). This heterogeneity underscores limits of one-size-fits-all diagnostics and supports personalized postprandial monitoring, including CGM-guided dietary interventions that outperform standard advice in randomized trials (45, 46).

#### 2.6.4 Inflammatory disruption of insulin sensitivity

Repeated exposure to saturated fats and refined carbohydrates activates pro-inflammatory kinases— IκB kinase beta (IKKβ) and c-Jun N-terminal kinase 1 (JNK-1)—that serine-phosphorylate IRS-1, impairing tyrosine phosphorylation and PI3K recruitment (preclinical) (39, 40). This molecular injury contributes to selective insulin resistance, where metabolic signaling decreases while inflammatory/lipogenic pathways remain active.

#### 2.6.5 Tissue-specific insulin resistance

Consequences differ by tissue. Skeletal muscle (approximately 80 percent of postprandial glucose disposal) develops GLUT4 translocation defects that limit uptake (47–49); the liver maintains gluconeogenesis/glycogenolysis despite hyperinsulinemia, sustaining hyperglycemia; and adipose tissue insulin resistance augments lipolysis and circulating FFAs, further propagating insulin resistance across organs (50).

### 2.7 Postprandial lipemia, triglyceride clearance, and lipotoxicity

#### 2.7.1 Normal postprandial triglyceride processing

After a mixed meal, dietary triglycerides are assembled into intestinal chylomicrons (CM) and reach the bloodstream via lymph within 30–60 min. Clearance depends on LPL and its endothelial anchor GPIHBP1 at capillaries of adipose tissue and skeletal muscle, enabling efficient intravascular hydrolysis and tissue uptake (7, 9, 51).

#### 2.7.2 Insulin-mediated regulation of lipid clearance

Physiologic postprandial insulin acutely increases LPL activity (e.g., post-heparin LPL) and promotes LPL trans-endothelial positioning via GPIHBP1 (52–56). Structural features of GPIHBP1 that accelerate LPL capture and luminal presentation have been defined (preclinical/biophysical) (53, 54). In insulin-sensitive states, this coordination rapidly hydrolyzes CM triglycerides, yielding controlled rises in tissue FFAs and minimal TRL remnants, with most clearance completed by approximately 2–4 h (55, 56).

#### 2.7.3 Pathological disruption of TRL metabolism

Insulin resistance lowers adipose LPL expression and raises endogenous LPL inhibitors— angiopoietin-like protein 3/angiopoietin-like protein 4 (ANGPTL3/ANGPTL4) and apolipoprotein C3 (APOC3)—slowing TRL hydrolysis and extending the lipemic phase from approximately 4–6 h to 8–12 h or longer, thereby sustaining exposure to atherogenic remnant particles (57–59).

#### 2.7.4 Vascular consequences of remnant accumulation

Small TRL remnants penetrate and are retained within the arterial intima (60, 61). They can be taken up by intimal macrophages—promoting foam-cell formation—and amplify chemokine/cytokine production, leukocyte adhesion, and vascular inflammation. This remnant-driven process contributes to residual atherosclerotic cardiovascular disease (ASCVD) risk beyond LDL-C lowering (61, 62).

#### 2.7.5 Hepatic lipid overload and MASLD progression

Elevated postprandial triglycerides create a “dual-TRL hit” to the liver: increased FFA influx (from impaired peripheral clearance and heightened lipolysis) fosters re-esterification and VLDL secretion, while CM remnants add lipid/cholesterol cargo. Together these inputs magnify dyslipidemia and drive hepatic steatosis and progression toward MASLD (63, 64).

#### 2.7.6 Cellular lipotoxicity and metabolic dysfunction

Excess FFAs generate diacylglycerol and ceramides that activate novel protein kinase C (PKC) isoforms, disrupt IR/IRS phosphorylation, and impair GLUT4 translocation, producing metabolic inflexibility with reduced glucose uptake and sustained hyperglycemia (65, 66).

#### 2.7.7 Inflammatory amplification and clinical biomarkers

Oxidized remnants and FFAs stimulate toll-like receptor 4 (TLR4), driving nuclear factor kappa-light-chain-enhancer of activated B cells (NF-*κ*B) signaling, upregulate NOX2/NOX4, and elevate IL-6 and tumor necrosis factor-alpha (TNF-α), while IL-1β can rise via inflammasome activation. In population settings, triglyceride incremental area under the curve (iAUC) > 5 mmol·h·L^−1^ associates with approximately 25 percent higher IL-6 within 4 h, supporting this metric as a prognostic marker of lipemic–inflammatory burden (67, 68). This environment decreases the bioavailability of endothelial NO and reinforces insulin resistance, closing the pathophysiological cycle (66, 69).

#### 2.7.8 Targeted therapeutic approaches

APOC3 antisense/siRNA accelerate CM and VLDL clearance, lowering peak postprandial triglycerides by up to approximately 45 percent in controlled trials (70, 71). ANGPTL3 inhibition (and to a lesser extent ANGPTL4) relieves LPL suppression, enhancing triglycerides hydrolysis and reducing remnants (72). Fibroblast growth factor 21 analogs improve hepatic β-oxidation and lower VLDL output, showing promise for MASLD-associated dyslipidemia (73).

### 2.8 Oxidative and mitochondrial stress in the postprandial window

A meal is more than caloric delivery—it is an acute redox challenge. Min after absorption, mitochondrial and enzymatic sources of ROS stimulate and briefly overcome endogenous antioxidant defenses. In healthy individuals this transient “spark” is hormetic, fine-tuning insulin action and vascular tone; in insulin-resistant or metabolic-syndrome phenotypes, the ROS pulse is higher and longer, synergizing with hyperglycemia and chylomicronemia to oxidize lipids/proteins, quench endothelial NO, and activate inflammasome/kinase pathways, feeding forward into endothelial dysfunction and insulin resistance (74, 75). Clinically, high-fat mixed meals reduce brachial-artery flow-mediated dilation (FMD) by approximately 1 percentage point at 2–4 h, placing peak vascular impairment squarely in the 60–180 min postprandial window (76). A concise mapping of sequelae, mechanisms, timing and read-outs is provided in Table 1.

**Table 1:** Oxidative-stress sequelae and clinical read-outs.

Consequence	Core mechanistic driver (concise)	Peak window post-meal (typical)	Primary read-outs (preferred)	Assay notes/standardization
Endothelial dysfunction	Superoxide reacts with NO to generate peroxynitrite; eNOS uncoupling from tetrahydrobiopterin depletion; NOX2/XO-derived ROS (77, 78).	1–4 h	Brachial-artery FMD (% change vs. baseline); plasma nitrite/nitrate; sNOX2-dp (if available)	Control caffeine/smoking and cuff/segment; adjust for baseline diameter; typical FMD drop ≈1 percentage point at 2–4 h
Inflammasome/innate immune activation	ROS activate IKKβ/JNK; these kinases recruit and oligomerize the NLRP3, which then activates caspase-1 (79, 80).	2–6 h	Plasma IL-1β and IL-18	Exclude acute infection; standardize timing and pre-analytical handling; freeze–thaw affects cytokines
β-cell stress and loss	Persistent ROS oxidize ER chaperones, forcing prolonged unfolded-protein-response signaling through PERK and eIF2α; the downstream rise in CHOP and caspase-3 expression accelerates pancreatic β-cell apoptosis (81).	Hours–days (magnified with repeated loads)	Proinsulin-insulin ratio (clinical proxy)	No direct plasma marker of β-cell apoptosis; interpret with glucose/FFA

NO, nitric oxide; eNOS, endothelial nitric-oxide synthase; NOX, NADPH-oxidase; FMD, flow-mediated dilation; ROS, reactive oxygen species; IKKβ, inhibitor-of-κB-kinase-β; JNK, c-Jun N-terminal kinase; NLRP3, NOD-, LRR- and pyrin-domain–containing protein 3; IL, interleukin; PERK, protein-kinase R–like ER kinase; eIF2α, eukaryotic initiation factor 2α; CHOP, C/EBP homologous protein (pro-apoptotic factor); FFA, free fatty acids.

Standardization details for platforms and pre-analytical handling are summarized in Supplementary Table S1.

Mechanistically, rapid substrate overflow raises the nicotinamide adenine dinucleotide (NADH:NAD^+^) and flavin adenine dinucleotide, reduced: flavin adenine dinucleotide (FADH₂:FAD) ratios, hyper-reduces CoQ, and favors RET at Complex I—an efficient *in vivo* superoxide source—while Complex III contributes under high membrane potential (82, 83). Parallel nutrient cues (acute hyperglycemia; TRL remnants) activate PKC—especially PKC-β—driving p47^phox translocation and NOX2/NOX4 activation; tetrahydrobiopterin depletion uncouples eNOS, and xanthine oxidase adds to ROS supply—together producing a convergent, multi-organ burst that typically peaks at 1–3 h (84–87).

In healthy muscle and endothelium, the postprandial ROS burst is normally self-limited by nuclear factor erythroid 2–related factor 2 (Nrf2)–driven induction of glutathione peroxidase, catalase, and heme oxygenase-1 (88–91). In metabolic-syndrome/MASLD phenotypes, Nrf2 tone and circulating antioxidants (e.g., bilirubin, paraoxonase-1) are diminished, shifting the balance toward peroxynitrite formation, LDL oxidation, and redox-sensitive inflammatory signaling (92–94). With repeated meals, unresolved redox stress extends beyond the 1–3 h window and engages β-cell unfolded-protein-response pathways [protein-kinase R–like ER kinase (PERK); eukaryotic initiation factor 2α eIF2α]), driving CHOP/caspase-3 and increasing vulnerability to apoptosis. There is no direct plasma marker of β-cell death; in practice, an elevated proinsulin:insulin ratio serves as a crude stress proxy (81).

Human translational data strengthen causality: reducing mitochondrial oxidants alleviates lipid-induced muscle insulin resistance, and postprandial metabolomics consistently show acylcarnitine signatures compatible with mitochondrial redox pressure and PDH inhibition during mixed-meal challenges (95, 96).

#### 2.8.1 Translational clues from intervention trials

Superoxide reacts with endothelial NO to generate peroxynitrite; the associated NO loss aligns with an approximately 1 absolute percentage-point decrement in brachial-artery FMD at 2–4 h after a single high-fat meal (76–78). ROS also signal through IKKβ/JNK to promote NLRP3 inflammasome assembly, increasing IL-1β and IL-18 (79, 80).

– **Polyphenols boluses.** Acute, meal-time polyphenols attenuate oxidative stress and can preserve endothelial function in standardized high-fat challenges (97). Examples include: (i) grape-seed extract taken 1 h pre-meal lowered oxide LDL and glucose exposure without changing insulin (metabolic-syndrome adults; *n* = 12; approximately 670 kcal mixed meal, approximately 40 percent fat/ approximately 50 percent carbohydrate; sampling 0–5 h) (98); (ii) grape-pomace extract co-ingested with a high-fat meal modulated oxidative-stress biomarkers with body mass index-stratified effects (healthy women; *n* = 18; 1,131 kcal high-fat meal, 66.7 percent fat; sampling 0–6 h) (99); and (iii) a high-flavanol cocoa beverage [150 mg (−)-epicatechin] co-ingested with a high-fat load preserved FMD during a standardized ischemia–reperfusion stress paradigm vs. a low-flavanol control (young healthy adults; *n* = 23; high-fat meal with 56.5 g fat; FMD assessed approximately 1.5–3.0 h post-meal) (100).
– **Targeting NADPH-oxidases.** Setanaxib (a selective NOX1/4 inhibitor) shows clinical signals in primary biliary cholangitis; postprandial vascular-endpoint trials (e.g., FMD, carotid-femoral pulse wave velocity) are still needed (primary biliary cholangitis; adults; randomized, placebo-controlled phase 2; *n* = 111; no test meal; 24 weeks; primary endpoint = %ΔGGT; secondary = ALP, liver stiffness, fatigue; vascular endpoints not assessed) (69).
– **N-acetylcysteine (NAC).** A single-blinded, placebo-controlled crossover in hypertensive adults showed that oral NAC (600 mg) reduced thiolated albumin (Thio-HSA) by approximately 25 percent at 60 min and i.v. NAC lowered it by approximately 69 percent at 30 min, with increased plasma antioxidant capacity—supporting rapid *in vivo* mercaptoalbumin regeneration relevant to the 0–3 h postprandial window (no meal challenge) (hypertensive adults; *n* = 6; oral or i.v. NAC; sampling 0–6 h) (84).
– **Mitochondria-targeted antioxidants.** During lipid/heparin infusion clamps (not a meal), intravenous mitoquinone increased insulin-stimulated leg glucose uptake and reduced ex vivo mitochondrial H₂O₂ emission in adult humans, directly linking mitochondrial oxidants to insulin resistance [adults; *n* = 10 (mitoquinone arm *n* = 9); 3 h intravenous lipid infusion + hyperinsulinemic–isoglycemic clamp; leg glucose uptake and muscle respirometry assessed approximately 30–120 min] (96).

Taken together, exaggerated ROS generation paired with an insufficient antioxidant response creates a modifiable hinge between nutrient overload and downstream vascular–metabolic injury. Curbing ROS production (e.g., NADPH-oxidase blockade, improved mitochondrial efficiency) or reinforcing endogenous defenses (Nrf2 activators, thiol donors, polyphenol-rich foods) may help re-establish the brief, adaptive nature of the postprandial redox signal (74, 75).

**Note.** Dedicated postprandial RCTs with vascular endpoints are lacking; current evidence supports a mechanistic, rapid thiol-replenishing action of NAC that is plausibly relevant to the 0–3 h window (84).

### 2.9 Endothelial activation and vascular inflammation

The vascular endothelium is the first interface to encounter postprandial blood. Under physiological conditions it releases NO, maintains an antithrombotic surface, and regulates nutrient delivery. Within min of a mixed meal, concurrent exposure to glucose, CM remnants, FFA, gut-derived lipopolysaccharide (LPS), and a burst of ROS can shift this interface toward a pro-inflammatory, vasoconstrictive phenotype—a shift amplified in obesity, MASLD, and chronic kidney disease (101, 102).

– **Mineral stress and calciprotein particles (CPP).** In chronic kidney disease, calcium–phosphate nanocrystals coated with fetuin-A circulate as CPPs. These colloids bind TLR4 on endothelial cells, activate NF-κB, upregulate VCAM-1/ICAM-1, and suppress eNOS phosphorylation; intravenous CPPs reproduce this injury pattern in ApoE-knockout mice (preclinical), underscoring systemic vasculotoxicity (103).
– **Canonical cytokine signaling.** TNF-α, IL-1β, and IL-6 converge on endothelial NOX2/NOX4, raising superoxide and uncoupling eNOS, thereby reducing bioavailable NO and impairing vasodilation. In meal tests, postprandial FMD falls within approximately 2–4 h (magnitude protocol-dependent) (104, 105).
– **Renin–angiotensin–YAP/TEAD crosstalk.** Angiotensin II activates YAP; nuclear YAP partners with TEAD factors to drive a VCAM-1 promoter, sustaining leukocyte adhesion. Verteporfin (YAP–TEAD disruptor) or endothelial YAP knockdown restores FMD and lowers VCAM-1 in mouse models (preclinical), highlighting a druggable redox-sensitive switch (106).
– **Gut–vascular signaling.** Metabolic endotoxemia (chronically elevated LPS from increased intestinal permeability) engages endothelial TLR4, boosts ROS, and further uncouples eNOS; TLR4 antagonism or antioxidant therapy rescues NO signaling and barrier integrity in cell and animal models (preclinical), linking dysbiosis to vascular dysfunction (107).

#### 2.9.1 Clinical snapshots

– **Obesity:** postprandial endothelial impairment is exaggerated; miR-485 mimics suppress NOX4, lower VCAM-1, and improve FMD in obese mice (preclinical) (108).
– **Human NOX2 signal:** high-fat meals provoke a rapid NOX2-dependent ROS burst; intravenous NOX2 blockade or a polyphenol-rich beverage at mealtime preserves endothelial function despite lipid load (human/challenge) (78, 104).

Oxidative–inflammatory endothelial injury sits at the crossroads of mineral imbalance, intestinal dysbiosis, systemic cytokines, and classic cardiometabolic risk. Limiting ROS generation (e.g., NOX inhibitors, improved mitochondrial efficiency), disrupting maladaptive transcriptional responses (YAP, NF-*κ*B), and reinforcing NO signaling may complement lipid- and blood-pressure–lowering strategies in restoring vascular health.

### 2.10 Inflammation and innate-immune activation

A mixed meal elicits a rapid innate-immune pulse (0–6 h): gut-derived LPS and other danger signals reach the portal circulation within 30–60 min, priming monocytes/macrophages. In metabolically healthy adults the surge resolves quickly; proinflammatory baseline, genetic liability, or frequent energy-dense meals amplify and prolong the response (109, 110).

– **Innate sensors and cytokine kinetics.** The NLRP3 inflammasome is a key nutrient-danger hub: a phosphate-enriched breakfast doubles caspase-1 activity in human monocytes and elevates IL-1β/IL-6 with approximately 2 h peak that wanes by approximately 6 h (111). TLR4 is activated by saturated fatty acids and CM remnants, driving dependent upregulation of VCAM-1 and ICAM-1, mechanistically linking dyslipidemia to endothelial dysfunction (110). Outside the vasculature, a fat bolus triggers hypothalamic astrocyte swelling and microglial activation by approximately 4 h in mice (preclinical) (112).
– **Inter-individual variation.** Host phenotype shapes the wave's height/duration: older adults with cardiometabolic risk generate approximately 40 percent higher peaks in IL-1β, C-reactive protein (CRP), and soluble ICAM-1 than healthy peers (113). APOE *ε*4 carriers mount approximately 2× postprandial rises in CRP/endothelial-activation markers vs. *ε*3/ε3 (114). Monocyte-subset dynamics also differ: CD14^+^^+^CD16^+^^+^; cells persist at 4 h in older adults but contract by approximately 50 percent in younger adults—evidence of innate-immune “memory” with aging (115).
– **Microbiome modulation and trained immunity.** Lower butyrate output is associated with sharper IL-6 and glycosylated acute-phase reactants (GlycA) peaks, an effect magnified by variants in sodium-coupled monocarboxylate transporter 1 [solute carrier Family 5 Member 8 (SLC5A8) or free fatty acid receptors 2 and 3 (FFAR2/3)] (116). Beyond innate signals, a single high-fat/high-sugar challenge can remodel T-cell chromatin at NF-κB– signal transducer and activator of transcription motifs and increase IL-17A for ≥1 week, consistent with diet-induced trained immunity (117).
– **Implications and levers.** The amplified cytokine environment accelerates vascular injury, upregulates endothelial adhesion molecules, and drives insulin resistance. Practical levers include boosting butyrate (resistant starch, inulin-type fructans), tempering TLR4 signaling (marine omega-3 fatty acids), and inhibiting NLRP3/ROS sources (mitochondrial antioxidants or NOX2 inhibitors), alongside post-meal physical-activity “snacks”.

### 2.11 Gut microbiota–derived signals in post-meal metabolism

During a mixed meal, host nutrients surge systemically while unabsorbed carbohydrate/protein reach the colon, where microbes generate SCFAs, secondary bile acids, and indoles that enter the portal vein near-synchronously with host substrates. Microbiome features (*α*-diversity; *Bacteroides/Prevotella/Akkermansia*) explain substantial between-person variance in postprandial glycemic and lipemic responses (systematic review of 36 trials; deep-phenotyping cohort *n* = 1,098) (118, 119).

– **Bile acid–L-cell axis (preclinical—human association).** High BSH activity rapidly deconjugates meal bile acids, increasing ligands for TGR5 on L-cells; in gnotobiotic mice, TGR5 blockade abrogates the GLP-1 surge and its glycemic benefit (preclinical) (120, 121). In humans, intestinal TGR5 messenger ribonucleic acid together with fecal BSH activity correlates with GLP-1 dynamics, explaining a meaningful fraction of 2-h GLP-1 iAUC variability (38, 67).
– **TRL output and clearance (preclinical with human links).** Antibiotic-treated mice show a approximately 35 percent fall in postprandial CM triglycerides paralleling reduced microsomal triglycerides and apoB-48 transcripts; *Bacteroides thetaiotaomicron* recolonization restores both expression and lipemia (preclinical). Indole-acetate suppresses ANGPTL4 via AhR, relieving the LPL brake and accelerating remnant clearance; conversely, microbial stimulation of GLP-2 upregulates enterocyte MTP/apoB-48, doubling CM output—effects blunted by a GLP-2R antagonist (preclinical) (38, 122).
– **Endocannabinoid and neuro-immune loops (human/preclinical).** In a randomized cross-over study, 2 h rises in N-acylethanolamines (e.g., anandamide, oleoylethanolamide) varied inversely with *Fecalibacterium*, with approximately 40 percent larger surges in metabolic syndrome (human) (123). Microbiota enriched in Enterobacteriaceae associate with sharper IL-6/IL-1β peaks and greater fullness after a Western-style meal, suggesting a gut–brain–immune loop (human association) (118). The lipid-lowering effect of endogenous GLP-1 depends on intact vagal afferents and is attenuated by acute fructose, implying neuroendocrine gating of CM handling (preclinical/physiology) (124).
– **Microbiota enriched.** In Enterobacteriaceae associate with sharper IL-6/IL-1β peaks and greater fullness after a Western-style meal, suggesting a gut–brain–immune loop (human association) (118). The lipid-lowering effect of endogenous GLP-1 depends on intact vagal afferents and is attenuated by acute fructose, implying neuroendocrine gating of CM handling (preclinical/physiology) (124).

**Note.** Evidence type for each circuit is indicated above; key models/readouts are summarized in Supplementary Table S1 (human vs. preclinical).

### 2.12 Integrative modulators of postprandial metabolism

Post-meal fuel handling emerges from the intersection of cellular energy sensors, multi-organ nutrient sensing, the microbiome, circadian clocks, and adipose–endocrine–neural feedback. These axes determine whether calories are oxidized, stored, or routed to gluconeogenesis/lipogenesis—helping explain person-to-person heterogeneity in glycemic and lipemic excursions.

1) **Cellular energy sensors (AMPK–mTORC1– Sirtuin-1).** When ATP falls, AMPK restrains mTORC1 and shifts flux toward fatty-acid oxidation/autophagy; higher NAD^+^/NADH activates sirtuin 1 (SIRT1), deacetylating PGC-1α/FOXO to support mitochondrial biogenesis and antioxidant defense. In the fed state, Akt re-engages mTORC1 to promote anabolism. Disrupting this AMPK–mTOR–SIRT1 switch accelerates steatosis, endothelial dysfunction, and insulin resistance (125, 126).
2) **Epithelial/host-context modulation.** In the intestinal epithelium, selective repression of IRS–PI3K–Akt drives FOXO nuclear entry, tightens junctions, lowers paracellular permeability, and can lower systemic triglycerides/glucose; hyperactivation does the opposite (preclinical) (127). Host factors further rewire this axis: SARS-CoV-2 proteins perturb IRS adaptors and upregulate suppressor of cytokine signaling-3, blunting Akt and contributing to *de novo* insulin resistance (human mechanistic/observational), while estrogen receptor-α scaffolds IRS-1 to bolster Akt–mTORC2 (mechanistic; sex-difference context) (128, 129). With chronic hypoinsulinemia (type 1 diabetes), liver IRS-2 falls as muscle AMPK/SIRT1 compensates—an adaptive multi-omics “rewiring” (130).
3) **Multi-organ nutrient sensing (gut–brain–pancreas).** Hypothalamic glucose-responsive neurons (GLUT2/ATP-sensitive potassium channel) and carnitine palmitoyltransferase 1C-positive neurons sense sugars and long-chain acyl-CoAs; L-cells convert luminal nutrient signals (SGLT-1; FFAR1/4; GPR119) into GLP-1/GIP/Peptide YY that reach the brainstem via vagal afferents. Vagotomy or acute fructose attenuates GLP-1–mediated suppression of CM triglycerides by approximately 35 percent, illustrating gut–brain control of postprandial lipemia (124). Microbial butyrate/indoles further tune this pathway (preclinical) (131).
4) **Circadian timing.** Core clock genes (brain and muscle ARNT-Like (BMAL1), circadian locomotor output cycles kaput (CLOCK), Period (PER) and cryptochrome (CRY)) gate insulin sensitivity and substrate partitioning. Front-loading energy at breakfast advances clock phase and blunts glucose/ triglycerides excursions, whereas the same load at dinner does the opposite; “Big-Breakfast” RCTs show approximately 38 percent lower post-meal glucose and upregulated leukocyte CLOCK/BMAL1 (132–136). Diet-induced thermogenesis is higher mid-afternoon than late night (137). Hepatic clock disruption increases nocturnal glucose output; intestinal clocks modulate CM assembly, explaining higher night-lipemia in circadian misalignment; PER2-deficient β-cells lose first-phase insulin release (138–140).
5) **Adipose buffering and endocrine–neural feedback.** In insulin-sensitive states, microvascular recruitment + LPL + GLUT4 trap dietary fat in adipose triglycerides stores. First-degree relatives of patients with T2D show approximately 40 percent smaller adipose blood-flow rises and approximately 35 percent greater non-esterified fatty acids (NEFA) spillover during mixed meals (141–143). LDL-receptor/CD36 density, visceral fat, and daily moderate to vigorous physical activity (MVPA) explain much of the spread in TAG iAUCs (144, 145). Circadian cues modulate adipose clocks (132, 146). Brown adipose tissue (BAT) activation via low-protein ketogenic diets or bile-acid signaling flattens triglycerides peaks and raises thermogenesis (human/rodent) (147). With aging, senescent visceral adipocytes (IL-6/TNF-α) amplify hyperglycemia; time-restricted eating or NAD^+^ boosters can blunt this signature (148).
6) **Neuro-endocrine crosstalk.** Vagal afferents relay luminal glucose/lipid/stretch to the nucleus tractus solitarius; silencing delays satiation and the return of insulin/GLP-1 to baseline, while optogenetic GLP-1 cell activation triggers nodose firing within approximately 60 s (149, 150). Dopamine released in proportion to dietary glucose enhances GLP-1 signaling in adipose, suppressing lipolysis and limiting NEFA spillover (151). After bariatric surgery, muted glucagon counter-surges can produce late dumping hypoglycemia, revealing pancreas–brain vulnerability (152). Functional magnetic resonance imaging links oxyntomodulin/GIP to reward-circuit activity; their rapid post-meal rise tempers this signal—exaggerated by added sugars (153, 154). Chemogenetic data suggest the brain sets approximately 30 percent of basal glucose turnover, whereas the pancreas controls approximately 70 percent of postprandial disposal, underscoring gut–brain–pancreas control of iAUC spread (155, 156).

Derailments across these axes—AMPK–mTOR imbalance, mistimed meals, loss of butyrate-producing microbes, impaired adipose perfusion, or a sluggish incretin–vagal relay—tilt metabolism toward postprandial hyperglycemia and hypertriglyceridemia. Interventions that align feeding with circadian phase, expand SCFA production, activate GLP-1/GIP receptors, or deploy very-low-energy ketogenic therapy to boost BAT capacity (130, 138, 157) are rational complements to calorie restriction and exercise, and fit an endocrine-centric MASLD prevention paradigm (138).

## 3 Biomarkers and clinical assessment of postprandial dysmetabolism

A mixed-meal test or CGM best captures postprandial physiology but remains resource-intensive. In practice, clinicians use fasting surrogates that mirror post-meal dynamics. Among them, the triglyceride–glucose (TyG) index stands out for consistency, cost, and external validity across settings.

### 3.1 Traditional markers and TyG index

The TyG index is calculated from early-morning blood drawn by multiplying fasting triglycerides (milligrams per deciliter) by fasting glucose (milligrams per deciliter), dividing that product by two, and then taking the natural logarithm of the result (158).

– **Dynamic signal.** Higher fasting TyG predicts steeper 2 h glucose and triglyceride rises on standardized meal tests—outperforming homeostatic model assessment for insulin resistance (HOMA-IR) (159).
– **Outcomes.** Across large cohorts, elevated TyG associates with faster carotid intima-media thickness (IMT) progression, higher incident CVD, ischemic stroke, and events in cancer survivors; in premature coronary artery disease (CAD), TyG ≥ 8.8 flagged approximately 75 percent higher 5-year major adverse cardiovascular event (159–163).
– **Comparisons and special populations.** Case–control work shows TyG (AUROC approximately 0.78) beats non-HDL-C and TG/HDL-C for angiographic stenosis; for MASLD, triglycerides/HDL-C slightly edges TyG (AUROC 0.82 vs. 0.80) (164, 165). In pediatrics, TyG > 8.2 detected abnormal glucose tolerance with approximately 82 percent sensitivity (166). Visceral adiposity (not total fat) drives the TyG–post-meal triacylglycerol (TAG) link, while ≥150 min/week MVPA halves the slope—supporting TyG as a modifiable risk indicator. Pairing TyG with meal-challenge or CGM traces yields a low-cost, high-yield view of postprandial burden (167). Operational details for TyG sampling/units are summarized in Supplementary Table S1 (TS1).

### 3.2 Emerging biomarkers: metabolomic, inflammatory & endothelial panels

– **Metabolomics (LC–MS).** Mixed meals transiently raise saturated ceramides (C16:0, C18:0), the C18:0/C24:0 ratio, branched-chain *α*-keto acids, medium-chain acyl-carnitines, and indole-3-propionate. Prospective data and meta-reviews identify ceramide C18:0/C24:0—especially with TyG—as a strong composite predictor of ASCVD events and IR conversion. Run times are falling as ion-mobility separation and machine learning (ML)–assisted readouts shorten gradients and automate pattern recognition, with sub-30 min workflows reported in research settings (168, 169). Pre-analytical handling and panel composition are detailed in TS1.
– Inflammation/innate immunity. GlycA (Nuclear Magnetic Resonance) integrates acute-phase glycoproteins; along with cluster of differentiation 163 (sCD163) and calprotectin, it outperforms high-sensitivity C-reactive protein (hs-CRP) for low-grade inflammation and predicts metabolic syndrome and coronary calcification. In severe dysmetabolism, neutrophil extracellular traps-derived cell-free DNA and IL-6 trans-signaling rise and track with carotid remodeling and impaired FMD (170, 171). Assay timing and stability notes appear in TS1.
– **Endothelial activation.** Glycocalyx shedding yields soluble thrombomodulin (sTM) and von Willebrand factor (vWF); endothelial extracellular vesicles (ICAM-1^+^) and miRNAs (miR-126-3p, miR-210) correlate with IMT progression and FMD decline, and portend mortality in severe COVID-19, underscoring a shared redox–endothelial axis (172, 173).
– **Composite scores.** Meta-analyses show TyG, TyG/waist, and triglycerides/HDL-C outperform LDL-C for detecting coronary disease, particularly in obesity/MASLD; adding vWF or miR-126 to TyG can push c-statistic > 0.80, rivaling costlier omics (174).

#### 3.2.1 Implementation (pragmatic workflow)

• **Step 1—**Screen with TyG and, where visceral adiposity is obvious, the triglyceride-to-HDL-cholesterol ratio.
• **Step 2—**Stratify intermediate-risk patients with GlycA and endothelial-vesicle counts to unmask subclinical inflammation or glycocalyx injury.
• **Step 3—**Personalize very-high-risk cases with ceramide/oxylipin panels to guide intensified lipid-lowering, antioxidant, or anti-inflammatory therapy (169, 170, 172).

Cutoffs, sample handling, and standardization are summarized in TS1.

### 3.3 Functional tests and dynamic indices—“Rate-of-Change” phenotyping

Static fasting values miss how fast systems absorb a meal-induced perturbation. Four protocols translate lability into time constants or impulse ratios clinicians can interpret:

• **Cardiorespiratory coupling time constant linking heart rate to oxygen-consumption kinetics (*τ*_HR–V˙O₂).** In a ramp-cycle test approximately 45 min post-breakfast, a time constant > approximately 60 s tracks upper-tertile TyG and predicts lower aerobic power at 12 months (175).
• **Impulse-based Dynamical Strength Index (IB-DSI).** A single countermovement jumps at approximately 2 h post-meal: impulse/maximum voluntary contraction ≤ 0.60 flags blunted neuromuscular recovery and co-segregates with higher ceramide C18:0/C24:0 and triglycerides peaks (176).
• **Dynamic-Fit Index (DFI).** Bayesian state-space fit to dense capillary glucose/lipid sampling; lower DFI (more error-corrections/min) precedes the first fasting-glucose rise by approximately 2 years (177).
• **Diaphragm excursion on four-dimensional computed tomography (4-D CT).** Failure to augment excursion by ≥10 percent after a meal associates with visceral adiposity, higher TyG, and heavier TAG iAUC (178).

### 3.4 Clinical relevance—why dynamic biomarkers matter

Post-meal signals anticipate hard outcomes years before fasting markers drift. Microbiome-informed ML models explain approximately 40 percent of variance in 2-h glucose iAUC, doubling glucose-only models; in PREDICT-1, this approach outperformed hemoglobin A1c and TyG for predicting conversion to impaired glucose tolerance (156, 157, 179). In T2D with CAD, TRL-TAG AUC > 5 mmol·h·L^−1^ forecasts microalbuminuria and hs-IL-6 increases within 12 months. Population data show non-fasting TAG 175 mg/dl beats the fasting 150 mg/dl cut-off for CVD risk (68, 142, 180–182). Palm-oil challenges that elevate ceramide d18:1/24:0 also raise VCAM-1 overnight; glycomics identify a fucose-rich, sialic-acid–poor N-glycan profile that flags incident T2D independent of glucose or TyG (183–185).

Taken together, postprandial biomarkers —whether they are kinetic (τ_HR–V˙O₂, IB-DSI, DFI), molecular (ceramides, GlycA), or microbial (butyrate-producing taxa)—capture *how resilient* an individual is to a metabolic load. Their predictive value supports a tiered clinical strategy:

• **Step 1 Screen** with inexpensive composites (TyG, triglyceride-to-HDL-cholesterol ratio).
• **Step 2 Stratify** intermediate-risk patients using GlycA plus a simple functional test such as *τ*_HR–V˙O₂.
• **Step 3 Personalize** (ceramides/microbiome-guided diets). Shifting from static concentrations to rates of change enables earlier, targeted intervention—before vascular, renal, or *β*-cell damage accrues.

Across large cohorts, highest-vs.-lowest strata of two-hour post-meal glucose exposure, triglyceride-rich lipoprotein triacylglycerol exposure, the triglyceride–glucose index, the plasma ceramide C18:0/C24:0 ratio, and glycoprotein acetylation show consistent graded risk. TS1 lists assay methods, cut-offs, and timing windows for each biomarker.

## 4 Nutritional and lifestyle interventions

Restore a brief, adaptive postprandial response by: (i) lowering substrate surges (glucose/TRL-TAG), (ii) dampening oxidative–inflammatory signaling, and (iii) aligning timing with circadian biology. Dynamic triggers to escalate care are summarized at the end (see also Supplementary Table S1).

### 4.1 Mediterranean-style eating as a postprandial buffer

The Mediterranean dietary pattern—extra-virgin olive oil (EVOO), vegetables, legumes, whole grains, fish, and modest red-wine use—consistently lowers cardiometabolic events (186, 187) and blunts postprandial “turbulence”. In healthy men, a single Mediterranean-type meal preserved endothelial function and attenuated triglyceride excursions vs. a high–saturated-fat comparator (healthy men; *n* = 28; randomized crossover; Mediterranean-type meal vs. high–saturated-fat meal, 858–885 kcal, 51–57 g fat; FMD and lipids 0–4 h) (111). In overweight/obese older adults, a Mediterranean-like meal produced smaller TAG rises than a Western high-fat meal while IL-6 increased similarly across meals [overweight/obese older adults; *n* = 60; randomized crossover; isoenergetic meals approximately 1,000 kcal (approximately 4,200 kJ); sampling 0–5 h] (111).

**Fine-tuning within the Mediterranean framework.** Small, targeted adjustments amplify benefits:

– **Gene–diet interaction.** In coronary-artery patients carrying the minor G-allele at zinc finger protein 1 (ZPR1) rs964184, switching from low-fat to Mediterranean reduced post-meal TAG by approximately 0.31 mmol·L^−1^; non-carriers changed little (188).
– **Carbohydrate quality.** Within an isocaloric Mediterranean day, replacing refined starches with low-GI pulses and whole grains blunted postprandial glucose/insulin excursions during an 8-h mixed-meal tolerance test (high-cardiometabolic-risk adults; *n* = approximately 180; standardized breakfast and lunch; sampling 0–8 h) (189). In type 2 diabetes, two isocaloric “healthy” patterns (Mediterranean-multifactorial vs. MUFA-rich) elicited distinct postprandial lipid and lipoprotein-subfraction responses after standardized test meals (T2D adults; randomized; serial sampling over several hours) (189).
– **Exercise synergy.** Adding approximately 150 min/week of brisk walking to a Mediterranean prescription improved the lipoprotein subclass profile (lower fasting triglycerides and small dense LDL, with favorable shifts in VLDL/LDL subclasses) (metabolic-syndrome adults; *n* = 202; energy-reduced Mediterranean diet + physical-activity promotion vs. energy-unrestricted Mediterranean diet; fasting NMR profiling; no standardized test meal) (190).
– **Fat-quality swap.** Replacing saturated fat with monounsaturated fat shifted the postprandial metabolomic profile toward lower acylcarnitines and higher antioxidant-related signals compared with a saturated-fat pattern; low-fat, high-complex-carbohydrate (LFHCC) arms with/without omega-3 (n-3) showed distinct postprandial signatures as well (metabolic syndrome; *n* = 75; randomized, 12-week isoenergetic diets: high–saturated fat [HSFA] vs. high–monounsaturated fat [HMUFA] vs. LFHCC vs. LFHCC + n-3; standardized high-fat challenge; sampling 0–8 h [0, 4, 8 h]) (191).
– **Timing matters.** Early time-restricted variants (“Mediterranean breakfast front-loading”) further dampen TAG/glucose peaks and improve adipose clock-gene expression (132, 133).

Across diverse trials, head-to-head crossover work shows a Mediterranean day outperforms DASH for 4-h TAG (approximately −18 percent) and oxidized-LDL, and a 2024 meta-analysis of ≥18 randomized controlled trials confirms reductions in fasting and postprandial TAG across healthy, pre-diabetic, and T2D cohorts (192). Practically, earlier eating with a Mediterranean first meal, low-GI pulses in place of refined starches, EVOO/marine ω-3 instead of saturates, and daily brisk walking magnify innate buffering. Response is not one-size-fits-all: ZPR1 rs964184 carriers show larger lipemic drops, whereas late chronotypes or habitual breakfast-skippers lose much of the gain. A 2024 umbrella review reporting parallel improvements in pre-diabetes conversion rates reinforces the pattern as a versatile, first-line, timing-aware prescription (193, 194).

### 4.2 Meal-timing and chrononutrition—aligning food with the body clock

Crossover trials, CGM studies, and meta-analyses converge: front-loading energy in the morning and tapering evening carbohydrates blunts glycemic and lipemic excursions, whereas breakfast skipping or late high-GI dinners do the reverse (195).

– **Illustrative signals.** Skipping breakfast increases lunchtime and dinnertime glycemic excursions in type 2 diabetes, accompanied by higher glucagon and lower iGLP-1 despite identical subsequent meals (T2D adults; *n* = 22; randomized crossover; breakfast vs. no breakfast with isocaloric lunch/dinner approximately 700 kcal; sampling 0–3 h) (196, 197). Shifting the main meal earlier—specifically, an early dinner at 18:00 vs. 21:00—lowers 24 h mean glucose and increases next-morning fat oxidation at identical energy intake (healthy adults; *n* = 12; randomized crossover; isocaloric day with dinner timing 18:00 vs. 21:00; 24 h CGM and next-morning indirect calorimetry) (198). Across randomized crossover trials, identical carbohydrate loads elicit higher evening than morning glycemic responses, with no consistent differences in insulinemia (adults with overweight/T2D; *n* = 8 crossover trials; standardized high-GI meals approximately 500–700 kcal; postprandial AUCs over approximately 2–3 h) (199).
– **Chronotype matters.** A randomized crossover stratified by chronotype showed that a high-GI dinner produced larger 2 h glucose excursions in late chronotypes, whereas early chronotypes had a comparatively attenuated evening response (healthy university students; *n* = 45; high-GI meal: cereal bar + cornflakes + milk + pretzel; breakfast 07:00 vs. dinner 20:00; CGM 0–3 h) (200). A complementary trial likewise found greater postprandial glycemia at dinner than at breakfast with identical high- vs. low-GI test meals (healthy older adults; *n* = 34 per protocol; high- or low-GI meals served at breakfast vs. dinner; capillary glucose 0–3 h) (201).
– **Early time-restricted eating (eTRE).** A short early window reduced 24 h mean glucose and glycemic variability and increased fat utilization without weight loss (overweight adults; *n* = 11; randomized 4-day crossover; eTRE 08:00–14:00 vs. 08:00–20:00; all meals provided; 24-h CGM; companion respiratory-chamber study) (202, 203). In a tightly controlled inpatient protocol, concentrating intake early in the day improved glycemic control and reduced glycemic variability under standardized conditions (healthy adults; *n* = 16; early vs. extended eating window as above; CGM 24 h; mixed-meal test 0–4 h) (203).
– **Within-meal sequencing.** In T2D, a small whey preload flattens early glycemia: 15 g whey taken 10 min before breakfast reduced the 0–240 min glucose iAUC and increased insulin/GLP-1 (T2D adults; *n* = 18; randomized crossover; 15 g whey 10 min pre-meal; standardized mixed-meal tolerance test; plasma sampling 0–4 h) (204). Evidence in type 1 diabetes is more heterogeneous but generally supports early-phase attenuation without worsening late hypoglycemia when modest doses are used; small crossover studies report blunted 0–120 min excursions with 10–20 g protein given 10–15 min before the meal, with dose and insulin strategy determining late effects (T1D adults; *n* approximately 10–30 across studies; 10–20 g protein 10–15 min pre-meal; capillary/CGM sampling 0–2–4 h) (205, 206). Across controlled-feeding studies, starting the meal with protein or fat (“protein-first/fat-first”) consistently lowers early postprandial glucose vs. carbohydrate-first, without raising triglycerides in the same window (mixed-risk adults; multiple small RCTs/crossovers; mixed meals typically approximately 500–900 kcal; sampling 0–2–4 h) (207).

### 4.3 Macronutrient manipulation—quality over quantity

Meta-analytic and crossover evidence (2020–2025) highlights three levers:

– **Swap refined carbohydrates for Monounsaturated Fatty Acids/Polyunsaturated Fatty Acids (MUFA/PUFA).** Replacing approximately 10 percent of carbohydrate with monounsaturated/polyunsaturated fat reduces postprandial glucose AUC by approximately 12 percent (adults with mixed risk; umbrella meta-analysis of approximately 27 RCTs; standardized test meals approximately 500–800 kcal; sampling 0–2/4 h) (208).
– **Protein preload (“micro-pulses”).** A small protein dose before the meal blunts the early glucose rise; approximately 20 g whey taken approximately 15 min pre-meal lowers glucose iAUC by approximately 12 percent (T2D/healthy adults; randomized crossover; mixed meals approximately 600–700 kcal; sampling 0–2 h) (209–211).
– **Resistant starch (RS) and fermentable fiber.** RS4 (phosphorylated wheat) acutely lowers incremental insulin iAUC and attenuates the second-meal glucose peak, while RS2 (potato) over weeks reduces fasting glucose and free fatty acids with modest, context-dependent postprandial improvements; a practical intake range is 15–30 g/day (overweight adults; RS4: *n* = 15; two standardized high-carbohydrate meals ∼600–800 kcal; sampling 0–180 min; RS2: *n* = 19; 12-week randomized crossover; standardized mixed-meal test ∼600–800 kcal; sampling 0–300 min) (212–214).

Shift refined-starch calories toward EVOO, nuts, and marine ω-3s; consider a 10–20 g protein preload before high-carb meals; and build RS-rich sides to boost butyrate and curb postprandial endotoxemia. Combine with Section 3.2 timing tactics for drug-like smoothing without pharmacotherapy.

### 4.4 Dietary bioactives and polyphenols—rapid-response molecules

Plant-derived secondary metabolites can blunt oxidative, inflammatory, and metabolic surges within min; with sustained intake they also re-condition endothelial and Nrf2 defenses and remodel the microbiome.

– **Catechins** **+** **chlorogenic acids (acute, dose–response).** In two randomized studies in healthy men, co-ingestion of combined catechins/chlorogenic acids produced a graded reduction in early postprandial glycemia (150 and 300 mg vs. 0 mg), supporting a practical pre-meal “rapid-response” strategy (healthy men; randomized designs; cookie-/drink-based tolerance tests; capillary/plasma sampling up to approximately 2 h) (215).
– **Anthocyanin-rich red raspberries.** In adults with prediabetes/insulin resistance, test meals containing 0, 125, or 250 g red raspberries on separate days produced dose-dependent metabolite changes with improvements in postprandial glucose/insulin dynamics across the day (adults with prediabetes/insulin resistance; randomized crossover; three meals with 0/125/250 g frozen red raspberries; plasma metabolites and glycemia 0–8 h and again at 24 h) (216).
– **Epigallocatechin gallate (EGCG) and Nrf2 pathway (mechanistic/kinetic support).** A physiologically based kinetic model integrating human data predicts that colonic metabolites of EGCG (e.g., gallic acid, pyrogallol) can reach concentrations sufficient to activate Nrf2-regulated gene expression *in vivo*, providing a mechanistic rationale for antioxidant “pre-meal” strategies (model-based prediction; fasting and non-fasting scenarios evaluated) (217).
– **Curcumin (longer-term).** Meta-analysis of randomized trials shows curcumin supplementation (≈80–1,000 mg/day for ≥4 weeks) lowers fasting glucose and CRP and improves overall glycemic indices—consistent with attenuation of chronic postprandial stress across meals (mixed adult populations; multiple RCTs; no standardized test meal; outcomes over weeks to months) (218).

For acute control, an approximately 150–300 mg catechin/chlorogenic-acid mix taken with or shortly before a carbohydrate-rich meal can dampen early glycemic excursions (0–2 h). In carbohydrate-heavy contexts, adding anthocyanin-rich fruit portions (e.g., red raspberries) to the meal supports postprandial glucose handling across the subsequent 8–24 h. For sustained conditioning of redox and inflammatory tone, multi-week curcumin courses can complement dietary timing and macronutrient strategies (Sections 3.2–3.3) (215).

### 4.5 surgical nutrition windows—pre-operative “Metabolic Priming”

Pre-operative nutritional status predicts wound healing, length of stay, and long-term outcomes after bariatric procedures. Two elements are consistently actionable:

– **Micronutrient optimization.** Many candidates present with subclinical iron, vitamin D, or thiamine deficits; routine screening and targeted repletion are recommended to minimize postoperative deficiency-related morbidity (e.g., fatigue, hair loss), although precise effect sizes for symptom reduction remain heterogeneous across studies (219, 220).
– **Very-low-calorie diet (VLCD) and Enhanced Recovery After Surgery bundle.** A 2–4-week protein-sparing VLCD reduces liver volume by about 16–17 percent and improves operative conditions; when embedded within an ERABS pathway, programs typically report shorter length of stay (approximately 1–2 days) and fewer overall complications. (Adults with severe obesity; VLCD 2–4 weeks; ERABS multimodal pathways) (221).

Treat the month before metabolic surgery as leverage—screen and replete micronutrients, implement a short VLCD to debulk hepatic fat while preserving lean mass, and apply ERABS protocols to temper inflammation and accelerate recovery (222, 223).

### 4.6 Physical-activity “Snacks” & structured exercise —turning skeletal muscle into a second pancreas

Even brief muscle contractions stimulate GLUT4 translocation and LPL activation. Breaking up sitting with 2–5 min bouts of standing or light walking every 20–30 min lowers postprandial glucose and insulin vs. uninterrupted sitting (adults with and without T2D; k approximately 22 randomized trials; standardized mixed meals approximately 500–900 kcal; sampling 0–2–3–4 h) (224). In people with T2D, desk-work break protocols similarly reduce postprandial glycemia and several studies report concurrent decreases in postprandial triglycerides during standardized meal tests (T2D adults; systematic review of break-frequency interventions every approximately 20–30 min during mixed-meal challenges; sampling 0–2–4 h) (225). Timing also matters: walking performed after meals produces larger reductions in postprandial glucose than the same walking done before meals (adults with overweight/T2D; meta-analysis of randomized crossover trials; identical standardized meals approximately 500–700 kcal; sampling 0–2 h) (226, 227). For intensity, high-intensity interval exercise reduces postprandial glucose and insulin vs. control and can outperform matched-work moderate-intensity exercise (mixed-risk adults; multi-study meta-analysis; meal-based and glucose-load protocols; outcome windows 0–2–4 h) (228). In practice: (i) stand or stroll 2–3 min at least every 30 min; (ii) add a short, well-timed bout within the first 2 h after eating (e.g., approximately 10 min of moderate walking); and (iii) remember that timing often beats duration—activity placed soon after a meal yields a larger immediate metabolic payoff than a longer session done late at night (implementation guidance from contemporary reviews) (229–231). In practice, brief, well-timed bouts yield measurable acute benefits across diverse populations; Table 2 summarizes representative activity-snack prescriptions (2020–2025) and their immediate metabolic effects.

**Table 2:** Representative “activity-snack” prescriptions (2020–2025) and acute metabolic effects.

Study/population	Prescription—timing and structure	Principal acute metabolic effect(s)
Meta-analysis of randomized trials in adults with and without T2D (k ≈ 22; standardized mixed meals ≈500–900 kcal; sampling 0–2–3–4 h	Standing or light walking for 2–5 min every 20–30 min during a seated lab protocol (224).	Two-hour glucose iAUC decreased by ≈12% and insulin iAUC by ≈20% vs. uninterrupted sitting
Systematic review focused on adults with T2D (breaks during desk-type tasks; standardized mixed-meal challenges; sampling 0–2–4 h)	At least one brief standing or slow-step break about every 20–30 min while seated work continued (225).	Postprandial glucose iAUC fell by ≈15% and TAG iAUC by ≈10% compared with continuous sitting
Meta-analysis of randomized crossover trials comparing post-meal vs. pre-meal walking (adults with overweight/T2D; standardized meals ≈500–700 kcal; sampling 0–2 h)	Brisk walking (about 10–20 min) initiated within ≈30 min after meals vs. the same dose before meals (226).	Greater reduction in postprandial glucose when walking is performed after meals; supports timing-sensitive placement of short bouts

CGM, continuous-glucose monitoring; iAUC, incremental area under the curve; TAG, triacylglycerol.

## 5 Pharmacological and technological advances —shrinking the postprandial “Damage Window”

Over the last half-decade, the emphasis has shifted from fasting targets to how quickly therapies flatten post-meal spikes. In parallel, continuous glucose monitoring (CGM) and algorithmic feedback allow clinicians to match fast-acting tools to the meals that need them most.

### 5.1 Pharmacological approaches that act within two to four hours after a meal

– **Enteroendocrine mimetics and co-agonists (human evidence).** Oral semaglutide lowers postprandial glucose exposure and attenuates TRL–TAG responses in phase-III settings (T2D; pooled phase-III meal-test substudies/post-hoc; standardized mixed meals approximately 500–700 kcal; sampling 0–4 h) (232). Tirzepatide (GLP-1/GIP) achieves comparable glucose control with additional reductions in TRL measures (T2D; SURPASS meal-test substudies/post-hoc; standardized mixed meals approximately 500–700 kcal; sampling 0–4 h) (233).
– **Adjunct glucose “shuttlers” (human evidence).** Faster-aspart reaches systemic circulation earlier than conventional rapid analogs and improves early post-meal control with less late hypoglycemia in CGM cohorts (T1D/T2D; real-world CGM; ad-libitum meals; 0–4 h CGM windows) (232). A single pre-prandial dose of empagliflozin reduces the 0–2 h glucose excursion in randomized crossover designs (T2D adults; randomized crossover; 5–25 mg immediately pre-meal; standardized mixed meal approximately 500–700 kcal; sampling 0–2–4 h) (234).
– **Lipid-centric modulators (human evidence).** PCSK9 inhibition reduces postprandial remnant/TRL exposure when added to background statins (T2D or mixed dyslipidemia; randomized add-on; standardized fat-tolerance tests; sampling 0–4–6 h) (235).
– **Bile-acid signaling (preclinical).** The dual FXR/TGR5 agonist INT-767 lowers postprandial TAG in high-fat-diet models; translation to clinical endpoints is ongoing (preclinical; HFD mice; oral fat tolerance or mixed lipid challenges; sampling approximately 0–4–6 h) (236).
– **Gut-facing/dual-action tools (early human).** LEAP-2 analogues (ghrelin antagonism) show acute appetite suppression with blunted glucose peaks in first-in-human testing (early human; single/short-course dosing; standardized liquid meal or OGTT; sampling approximately 0–2–4 h) (237). Endoscopic duodenal devices (e.g., mucosal resurfacing or sleeves) improve postprandial glucose/insulin dynamics in early studies (pilot human plus DIO-rat support; standardized mixed meal; sampling approximately 0–2 h) (238).

Table 3 (unchanged in structure) summarizes acute mechanisms, magnitude where reported in your sources, and development stage for agents with 0–4 h post-meal impact—strictly aligned with refs (232, 234–238).

**Table 3:** Pharmacological agents that flatten the 0- to 4 h post-meal window: dominant acute mechanism, key efficacy data and development stage.

Class/agent(s)	Dominant acute post-meal effect	Key efficacy data (design & population)
Entero-endocrine mimetics/co-agonists Oral semaglutide -Tirzepatide	Semaglutide lowers 4-h glucose iAUC and TRL-TAG iAUC; tirzepatide achieves comparable glucose control with additional TRL reductions (where reported) (233, 239).	Phase-III T2D programs with meal-test substudies/post-hoc analyses; standardized mixed meals ≈500–700 kcal; sampling ≈0–4 h.
Adjunct glucose “shuttlers” Faster-aspart—Empagliflozin (pre-meal)	Faster-aspart reaches systemic circulation ≈10 min sooner than standard rapid analogs and reduces late hypoglycemia; single pre-prandial empagliflozin dose lowers early glucose excursion (232, 234).	Real-world CGM cohorts (T1D/T2D; ad-libitum meals; 0–4 h CGM windows) for faster-aspart. Randomized crossover (T2D adults; 5–25 mg immediately pre-meal; standardized mixed meal ≈500–700 kcal; sampling ≈0–2–4 h) for empagliflozin.
Lipid-centric modulators Alirocumab (anti-PCSK9) INT-767 (dual FXR/TGR5)	PCSK9 inhibition reduces remnant/TRL exposure post-prandially (human); INT-767 lowers TAG iAUC in HFD mice (preclinical) (235, 236).	Alirocumab: randomized add-on in T2D/mixed dyslipidemia; fat-tolerance/mixed-meal tests; sampling ≈0–4–6 h. INT-767: preclinical HFD mouse models; lipid challenge tests; sampling ≈0–4–6 h.
Gut-facing/dual-action tools LEAP-2 analog · Endoscopic duodenal sleeve	LEAP-2 analog blunts glucose peaks without hypoglycemia (early human); duodenal sleeve improves 2 h glucose/insulin responses (pilot human; DIO-rat support) (237, 238).	LEAP-2: first-in-human; standardized liquid meal/OGTT; sampling ≈0–2–4 h. Sleeve: DIO-rat plus pilot human; standardized mixed meal; sampling ≈0–2 h.

CGM, continuous-glucose monitoring; FIH, first-in-human; FXR, farnesoid X receptor; GIP, glucose-dependent insulinotropic polypeptide; HFD, high-fat diet; iAUC, incremental area-under-the-curve; PCSK9, pro-protein-convertase-subtilisin/kexin 9; TAG, triacylglycerol; TRL, triglyceride-rich lipoprotein; T2D, T2D; TGR5, takeda G-protein-coupled receptor 5.

### 5.2 Digital therapeutics and AI-assisted food coaching

CGM-guided, algorithm-predicted diets reduce time above range and blunt 0–2 h glucose rises in primary-care programs vs. general advice, with high adherence due to actionable, real-time nudges (45, 240). Integrating CGM into inpatient and outpatient workflows reduces glycemic variability and unmasks “silent” post-meal excursions that fasting tests miss (241–243). Personalized postprandial targeting menus informed by individual features (including microbiome signals) outperform standard patterns for several glycemic metrics in selected cohorts (155, 244).

### 5.3 Clinical implementation—linking postprandial control to liver health

In MASLD, attenuating post-meal glucose/TRL/oxidative surges is clinically relevant (245–247). A pragmatic sequence is:

1) Screen with TyG ± non-fasting TAG or a simple TRL-TAG curve;
2) prescribe a Mediterranean template with earlier energy distribution plus brief post-meal activity;
3) if high postprandial burden persists, escalate with GLP-1/GIP co-agonists or PCSK9 inhibitors;
4) repeat liver enzymes and a post-meal TAG assessment at approximately 12 weeks to adjust therapy

## 6 Conclusions and future directions

Postprandial metabolism is now recognized as a network of druggable nodes, extending from the gut lumen to the vascular wall. Three key targets are gaining traction: the enterohepatic bile acid loop, intracellular steroid and SUMO switches, and nutrient-sensing GPCRs. Promising agents already in development reduce mixed-meal triglycerides, reverse insulin resistance, and disrupt lipogenesis and late-phase hyperinsulinemia.

Importantly, these post-meal metabolic surges are not only cardiometabolic but also oncogenic triggers—fueling inflammation, insulin signaling, and epithelial dysplasia. Early shifts in glucose and triglyceride waves, impaired thermogenesis, and altered bile acid profiles are strong predictors of diabetes, fatty liver, and vascular damage—often before fasting markers change.

Advanced multi-omics, real-time wearables, and AI pipelines are transforming these insights into precision care. Emerging tools now outperform classical risk scores, identify distinct postprandial endotypes, and enable real-time interventions that significantly reduce glycemic exposure. As these technologies scale, equity-centered frameworks will be essential to ensure access, relevance, and impact across diverse populations.
