# Supplementary material for: From meal to malfunction: exploring molecular pathways, biomarkers and interventions in postprandial cardiometabolic health
Source: Front Cardiovasc Med. 2025 Oct 29;12:1655889. doi: 10.3389/fcvm.2025.1655889 (PMC12605470; doi:10.3389/fcvm.2025.1655889)
Supplement: Supplementary file 1 [file Table1.docx]

**Supplementary Table S1. Evidence anchors, assay standards, temporal bands, and evidence flags**

This multi-panel supplementary table summarizes: (A) pre‑2020 foundational anchors used strictly for background/mechanistic context; (B) assay and pre‑analytical standards with practical windows and reporting notes; (C) operational temporal bands for postprandial physiology and recommended sampling; and (D) evidence flags and how each category is applied in the review.

**Panel A. Foundational anchors (pre‑2020)**

Foundational anchors (pre‑2020) used to frame mechanisms, methods, and historical context. Each item maps to the manuscript reference number in brackets [Ref #].

| **Domain / topic** | **Foundational anchor (pre‑2020) [Ref #]** | **Study type** | **Key finding (≤1 line)** | **Rationale for inclusion** | **Limits / notes** | **How used in the review (restrictions)** | **Section(s) cited** |
| --- | --- | --- | --- | --- | --- | --- | --- |
| Non‑fasting triglycerides and cardiovascular risk | Guidance/meta‑analyses linking non‑fasting TAG to events [Ref 1.2.7: 66-69; 2.4: 68,143, 157,158,180–186] | Consensus / Meta‑analysis | Non‑fasting TAG better predicts events than fasting in general populations. | Anchor for using non‑fasting TAG and TRL measures. | Cut‑points vary by cohort; heterogeneity across assays. | Background rationale; not used to claim effect sizes for 2020–2025 results. | 2.4, 1.2.7, Panel B |
| Postprandial glucose iAUC and carotid intima‑media thickness | Prospective cohort linking meal glucose peaks to IMT progression [Ref Introduction: 1-23; 2.4: 68,143, 157,158,180–186] | Prospective cohort | Higher 2‑h glucose excursions predict IMT progression independent of fasting. | Justifies focus on dynamic, post‑meal metrics. | Older meal protocols; capillary sampling. | Framing only; contemporary estimates come from 2020–2025 studies. | Introduction, 2.4 |
| LPL–GPIHBP1 axis in TRL clearance | First biophysical descriptions of GPIHBP1‑mediated LPL shuttling [Ref 41-42] | Mechanistic / Biophysical | Defined endothelial presentation of LPL as a rate‑limiting TRL‑clearance step. | Mechanistic scaffold for Section 1.2.2. | Preclinical/biophysical; not clinical efficacy. | Mechanistic context only. | 1.2.2 |
| NADPH oxidase / eNOS uncoupling and endothelial function | Early work on NOX‑derived ROS and eNOS uncoupling in humans [Ref 1.3: 74-96] | Mechanistic / Translational | Postprandial ROS can quench NO and depress flow‑mediated dilation. | Explains timing of FMD nadir in 60–180 min window. | Small studies; varied protocols. | Mechanistic backdrop; current trials summarized in 1.3.1. | 1.3, 1.4, Panel C |
| NLRP3 inflammasome priming by nutrient signals | First‑in‑field studies on postprandial inflammasome activation [Ref 109-117] | Mechanistic / Human challenge | Acute nutrient loads can raise IL‑1β/IL‑18 via NLRP3. | Supports cytokine windows in 2–6 h band. | Assay handling critical for cytokines. | Mechanistic context only. | 1.5, Panel C |
| Circadian modulation of metabolic responses | Early human/clock‑gene and chrono‑nutrition studies [Ref 1.1.3: 43-46; 3.2: 197-209] | Mechanistic / Cohort / Crossover | Evening meals elicit larger glycemic/lipemic responses than morning meals. | Frames meal‑timing section and temporal bands. | Heterogeneous protocols and chronotype measures. | Background context; contemporary RCTs cited for effects. | 1.1.3, 3.2, Panel C |
| Mediterranean diet and cardiometabolic outcomes | Pivotal RCTs/meta‑analyses on Mediterranean patterns [Ref 111,187-196] | Pivotal RCT / Meta‑analysis | Mediterranean pattern reduces cardiometabolic events. | Establishes relevance of pattern used for postprandial buffering. | Event‑level outcomes; not meal‑test focused. | Context only; acute meal data from 2020–2025 crossovers. | 3.1 |
| GLP‑1 physiology (gut–brain–liver lipid control) | Foundational physiology of GLP‑1 effects on lipemia [Ref 1.7: 125,158; 4.1: 235-240] | Mechanistic / Physiology | GLP‑1 signaling can reduce chylomicron output and lipemia. | Supports incretin rationale in Sections 1.7 and 4.1. | Preclinical/physiology; context only. | Mechanistic support, not effect sizing. | 1.7, 4.1 |
| TyG index as insulin‑resistance proxy | Early validation cohorts of TyG [Ref 2.1: 159-168] | Cohort / Case‑control | TyG associates with insulin resistance and cardiometabolic risk. | Justifies TyG as a low‑cost screen. | Cut‑offs vary; unit conventions differ. | Screening rationale; cut‑offs contextualized in Panel B. | 2.1, Panel B |
| Bile acids–TGR5–L‑cell axis | First demonstrations linking microbial BSH, bile acids, and L‑cell secretion [Ref 1.7: 125,158; 67,128-125] | Preclinical / Human association | Microbial bile‑acid remodeling modulates GLP‑1 via TGR5. | Mechanistic scaffold for microbiome sections. | Species‑ and site‑specific effects. | Mechanistic context only. | 1.6, 1.7 |

**Panel B.** Assay & pre‑analytical standards (windows, practical cut‑offs, standardization notes)

| **Biomarker / test** | **What it captures (clinical intent)** | **Practical cut‑points / targets (contextual)** | **Sampling window & protocol** | **Pre‑analytical / assay notes** | **Preferred reporting format** |
| --- | --- | --- | --- | --- | --- |
| Triglyceride–glucose (TyG) index | Low‑cost fasting surrogate of postprandial burden and insulin resistance. | Report as continuous; example risk strata often ≈ 8.3–9.0; ≥8.8 flagged in premature CAD in manuscript. | Fasting morning sample; same lab units each time. | Formula: ln([fasting TG mg/dL × fasting glucose mg/dL]/2); unit conventions must be consistent. | Numerical value (2 decimals) + percentile or cohort‑specific quartile. |
| Non‑fasting triglycerides | Atherogenic exposure closer to real‑world eating; remnant risk. | ≥175 mg/dL (≈2.0 mmol/L) repeatedly non‑fasting suggests elevated risk (context‑dependent). | Sample 2–6 h post‑typical meal or random daytime non‑fasting. | Record time since last meal and meal type; avoid alcohol/acute illness. | Concentration with time‑since‑meal; consider median of multiple occasions. |
| TRL‑TAG iAUC (remnant burden) | Integrated chylomicron/VLDL remnant exposure after a mixed meal. | >5 mmol·h·L⁻¹ associates with higher IL‑6 rises within 4 h (manuscript context). | Standardized mixed meal (≈500–800 kcal); samples at 0, 2, 4 (±6) h. | Specify meal composition; use identical assay lots for serial testing. | iAUC with meal recipe, timepoints, and units. |
| Flow‑mediated dilation (FMD) | Conduit artery endothelial function; sensitive to postprandial oxidative stress. | Typical absolute drop ≈1 percentage point at 2–4 h after high‑fat meals. | Baseline and 2–4 h post‑meal (same arm, segment, cuff). | Caffeine/smoking abstinence; adjust for baseline diameter; trained sonographer. | Absolute % change from baseline (with CIs). |
| Continuous glucose monitoring (CGM) metrics | Dynamic glycemic exposure and variability across meals and day. | Lower time‑above‑range and glucose coefficient‑of‑variation; targets per guideline and phenotype. | 24‑h periods spanning meals; annotate meal times and composition. | Sensor calibration/lag; exclude compression artifacts; standardize meal logging. | TAR/TBR/TIR, mean glucose, CV%, post‑meal 0–2 h excursion. |
| GlycA (NMR) | Composite of circulating glycoprotein acetylation (low‑grade inflammation). | Interpret longitudinally; lab‑specific reference intervals. | Fasting or standardized non‑fasting; time‑of‑day consistency. | Same NMR platform; avoid acute infection; report units per platform. | Absolute value + change from baseline. |
| Ceramide ratio C18:0/C24:0 | Cardiometabolic risk enrichment; tracks adverse lipid remodeling. | Higher ratios associate with events; use lab‑specific centiles. | Fasting baseline for risk; optional post‑meal dynamics in studies. | LC–MS with internal standards; batch correction; hemolysis avoidance. | Ratio with platform/normalization details. |
| Endothelial extracellular vesicles (ICAM‑1⁺, etc.) | Endothelial activation/injury signal; relates to IMT/FMD. | No universal cut‑off; use within‑study tertiles or change from baseline. | Baseline and 2–4 h post‑meal in research settings. | Flow cytometry or NTA; pre‑analytical handling (freeze–thaw) critical. | Counts per μL and phenotype markers. |
| Cytokines (IL‑6, IL‑1β, hs‑CRP) | Innate immune activation; links to remnant/ROS burden. | Interpret peaks and deltas rather than single cut‑offs. | 0–4–6 h post standardized meal; avoid intercurrent infection. | Strict timing; chilled tubes; rapid spin; minimize freeze–thaw cycles. | Concentration vs time with iAUC where applicable. |

## Panel C. Operational temporal bands and recommended sampling

| **Temporal band** | **Dominant processes** | **Typical peaks (illustrative)** | **Recommended read‑outs** | **Suggested sampling time‑points** |
| --- | --- | --- | --- | --- |
| 0–60 min | Rapid glucose absorption; first‑phase insulin and incretin surge; autonomic changes. | Earliest glucose/insulin upswing; GLP‑1/GIP rise; gastric emptying effects. | Capillary/plasma glucose; insulin/C‑peptide; early incretins (research). | 0, 30, 60 min (meal start = 0). |
| 60–180 min | TRL appearance/expansion; mitochondrial/enzymatic ROS burst; FMD nadir. | FMD drop ≈ 1 percentage point at 2–4 h after high‑fat meals; IL‑6 begins to rise. | TAG/TRL metrics; FMD; nitrite/nitrate; oxidative markers; CGM 0–2 h. | 0, 120, 180 (±240) min. |
| 120–360 min | Remnant lipemia; endothelial activation (VCAM‑1/ICAM‑1); inflammasome signaling. | IL‑6 2–4 h; adhesion molecules 3–6 h; TRL remnants persist if clearance is slow. | TRL‑TAG iAUC; IL‑6; soluble ICAM‑1/VCAM‑1; endothelial EVs. | 0, 120, 240, 360 min (protocol‑dependent). |
| Hours–days | Microbiome‑endocrine adjustments; trained‑immunity signals; delayed vascular effects. | Overnight adhesion‑molecule changes; next‑day metabolic set‑points. | Next‑morning fasting panel; selected metabolomics; CGM day‑over‑day metrics. | Baseline, same‑day post‑meal, and next‑morning fasting. |

## Panel D. Evidence flags and how they are applied

| **Evidence flag** | **Definition** | **Allowed uses in the review** | **Not used for** | **How flagged in text** |
| --- | --- | --- | --- | --- |
| Foundational / pre‑2020 | Pre‑2020 anchors (meta‑analyses, consensus, pivotal RCTs, first‑in‑field mechanisms). | Historical framing, mechanistic scaffolding, methods/assay context. | Estimating clinical effect sizes or endpoints for 2020–2025 claims. | Explicitly tagged as “foundational” and listed in Supplementary Table S1 (Panel A). |
| Preclinical | Animal/cell models; pharmacology/mechanisms not tested in humans postprandially. | Causality in pathways; nominating druggable targets. | Clinical efficacy claims or effect‑size estimation. | Labeled “preclinical” with model and exposure type in‑text. |
| Human 2020–2025 | Adult human evidence (RCTs, challenge tests 0–6 h, prospective cohorts) within 2020–2025. | Primary quantitative support for magnitude/timing and practice statements. | Background mechanistic attributions beyond reported data. | Cited normally, with parenthetical who/meal/window descriptors. |
| Assay / Methods | Standardization papers, position statements, or protocols. | Defining sampling windows, handling, and reporting formats. | Outcome claims outside methodological scope. | Identified in Panel B and described in Methods/Notes in text. |
